# Concurrent surgical repair demonstrates favorable short-term clinical outcomes in patients with labral tears and concomitant hip rotator cuff injuries: a 2-year propensity score-matched cohort study

**DOI:** 10.3389/fspor.2026.1799902

**Published:** 2026-04-24

**Authors:** Haoling Li, Xinxin Lang, Yi Liu, Ziyi Lin, Xiangyu Yin, Shiyu Sha, Qingfeng Yin

**Affiliations:** 1 Department of Sports Medicine, The Second Qilu Hospital of Shandong University, Shandong University, Jinan, Shandong, China; 2 Department of Joint Surgery, PKUCare Luzhong Hospital, Zibo, Shandong, China

**Keywords:** acetabular labral repair, femoroacetabular impingement, hip, hip arthroscopy, hip rotator cuff injury

## Abstract

**Background:**

Acetabular labral tears and hip rotator cuff injuries frequently coexist and present with overlapping symptoms, posing diagnostic and therapeutic challenges. However, outcomes of simultaneous arthroscopic repair for these combined pathologies remain unclear. This study evaluated short-term outcomes after arthroscopic hip rotator cuff repair performed concurrently with acetabular labral repair vs. labral repair, while describing surgical considerations and characterizing the demographic and clinical features of concomitant injuries.

**Methods:**

In this retrospective matched-cohort study, patients who underwent primary arthroscopic concomitant repair of acetabular labral tears and hip rotator cuff injuries between September 2019 and September 2023 were identified. These patients were matched in a 1:2 ratio based on sex, age (±2 years), and body mass index (BMI, ±5 kg/m^2^) to patients who underwent arthroscopic labral repair alone. Patient-reported outcomes (PROs) were evaluated preoperatively and at 2-year follow-up, utilizing the modified Harris Hip Score (mHHS), Western Ontario and McMaster Universities Osteoarthritis Index (WOMAC), International Hip Outcome Tool–12 (iHOT-12), and Visual Analog Scale (VAS) for pain. Secondary outcomes included the proportions of patients achieving the minimal clinically important difference (MCID) and the patient acceptable symptom state (PASS).

**Results:**

Thirty-one patients underwent arthroscopic repair of hip rotator cuff injuries with concomitant labral repair (83.87% female; mean age, 52.13 ± 7.47 years; mean BMI, 28.33 ± 5.42), and were matched to 62 patients who underwent labral repair alone (83.87% female; mean age, 51.97 ± 7.21 years; mean BMI, 28.50 ± 5.35). Baseline characteristics, including age, sex, BMI, preoperative imaging findings, and preoperative PROs, were comparable between the groups (all *P* > 0.05). At the 2-year follow-up, both cohorts demonstrated significant improvement in all PROs compared with preoperative values (all *P* < 0.001), with no significant intergroup differences in postoperative PRO scores (all *P* > 0.05). The proportions of patients achieving the MCID and PASS were comparable between the groups (all *P* > 0.05).

**Conclusion:**

In patients undergoing arthroscopic hip rotator cuff repair with concomitant labral repair, no significant differences in short-term clinical outcomes were observed compared to those undergoing arthroscopic labral repair alone, indicating that simultaneous arthroscopic repair yields favorable short-term results.

## 1 Introduction

With the advancing understanding of hip joint disorders, acetabular labral tears have become a common clinical cause of hip pain and functional limitation in young and middle-aged adults. The prevalence of acetabular labral tears among patients presenting with anterior hip or groin pain has been reported to range from 22% to 55% (1). Meanwhile, emerging evidence indicates that hip rotator cuff injuries—primarily involving the tendons of the gluteus medius and gluteus minimus—are also an important and often overlooked source of hip pain in middle-aged and older populations, with a markedly increased incidence in individuals over 50 years of age (2). In clinical practice, patients with concomitant acetabular labral tears and hip rotator cuff injuries are not rare, and the symptoms and clinical presentations in these patients often overlap with those of only labral injury, which presents new challenges for diagnosis and treatment.

With the advancement and extensive adoption of hip arthroscopy, substantial clinical evidence has confirmed the efficacy of arthroscopic repair for isolated acetabular labral tears (3–5). Similarly, arthroscopic repair of the hip rotator cuff, referring to injuries of the gluteus medius and gluteus minimus tendons, has been demonstrated to effectively alleviate lateral hip pain and improve functional outcomes (6–8). Nonetheless, in routine clinical practice, it is not rare for acetabular labral tears to coexist with hip rotator cuff injuries. Although preliminary evidence suggests that simultaneous repair may lead to favorable clinical outcomes (9), the optimal surgical strategy for patients with both acetabular labral tears and hip rotator cuff injuries remains controversial. Ongoing debate persists regarding whether to perform staged procedures, prioritize intra-articular labral management, or undertake simultaneous repair of both labral tears and hip rotator cuff injuries. Meanwhile, the safety of simultaneous surgical repair of acetabular labral tears and hip rotator cuff injuries, as well as its impact on early functional recovery, requires further evaluation. Additionally, the clinical characteristics of this patient population and their associations with key factors such as sex, age, and pelvic morphology remain unclear.

Therefore, the purpose of this study was to evaluate the safety and short-term clinical outcomes of simultaneous arthroscopic repair for patients with acetabular labral tears and hip rotator cuff injuries. We hypothesized that simultaneous surgical repair of acetabular labral tears and hip rotator cuff injuries would not adversely affect early functional recovery and that its short-term clinical outcomes would be non-inferior to those of patients undergoing isolated labral repair. Furthermore, we aimed to characterize the epidemiological profile of this cohort and provide a detailed description of the surgical technique employed.

## 2 Methods

### 2.1 Patient selection

**Figure 1:**
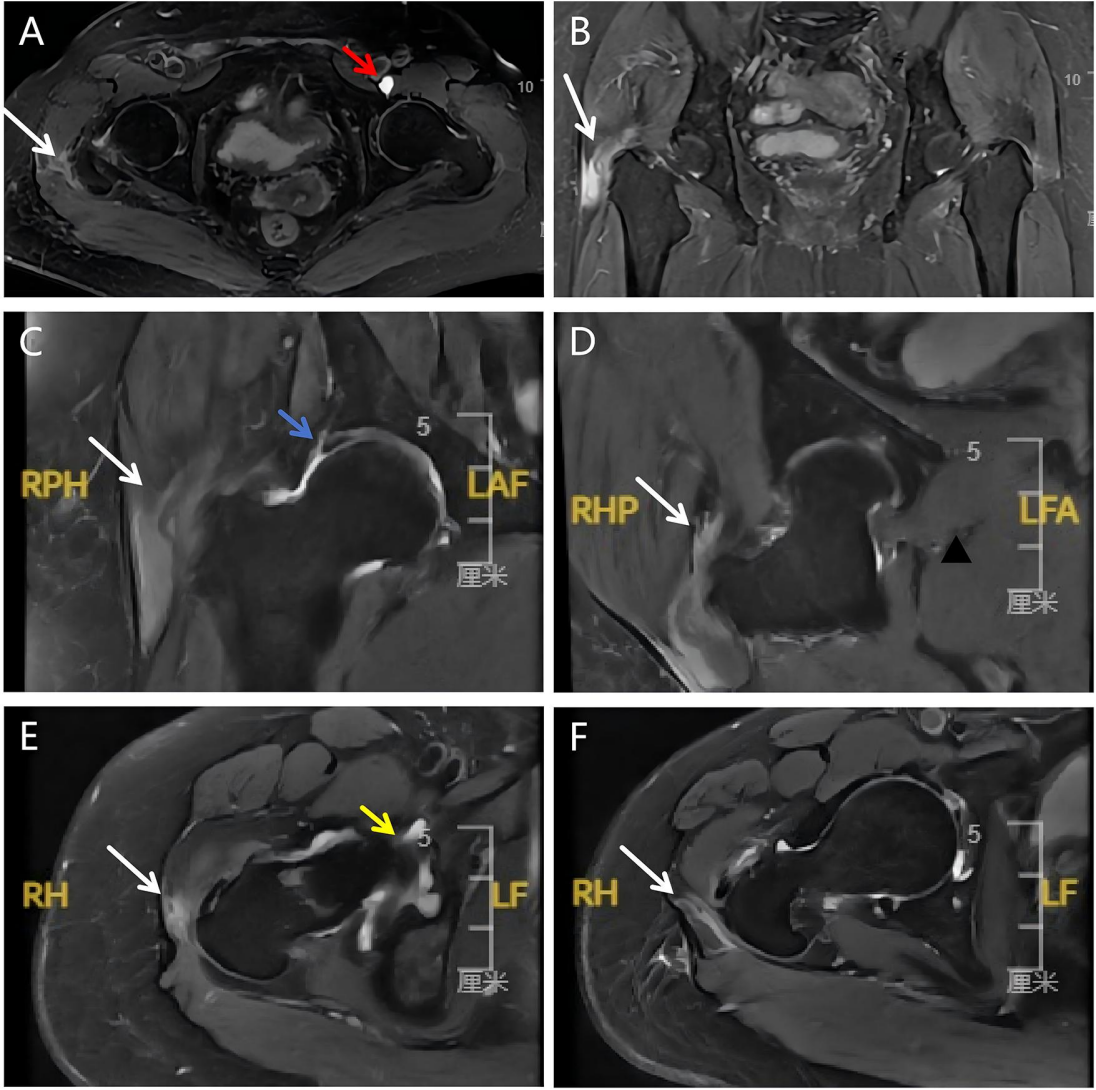
Representative MRI findings of hip rotator cuff injuries across different sequences and imaging planes. **(A)** Axial fat-suppressed MRI of both hips shows hyperintense signal around the right greater trochanter (white arrow), indicating a hip rotator cuff tear. A hyperintense labral cyst is present in the left hip (red arrow). **(B)** Coronal fat-suppressed MRI of both hips demonstrates marked hyperintensity around the greater trochanter (white arrow) with discontinuity of the gluteus medius tendon at its insertion, indicating a full-thickness tear. **(C,D)** Coronal and oblique coronal fat-suppressed MRI of the affected hip show heterogeneous signal at the gluteus medius insertion on the greater trochanter apex (white arrows), indicating a hip rotator cuff injuries. A linear hyperintense cleft between the acetabular labrum and acetabulum (blue arrow) suggests a labral tear. **(E)** Oblique sagittal fat-suppressed MRI of the hip demonstrates edema-like hyperintensity at the gluteus medius tendon insertion of the hip rotator cuff (white arrow), suggesting hip rotator cuff edema. Associated joint effusion is also present (yellow arrow). **(F)** Axial proton density–weighted fat-suppressed MRI shows hyperintensity at the gluteus minimus tendon insertion (white arrow), consistent with a gluteus minimus tendon tear.

**Figure 2:**
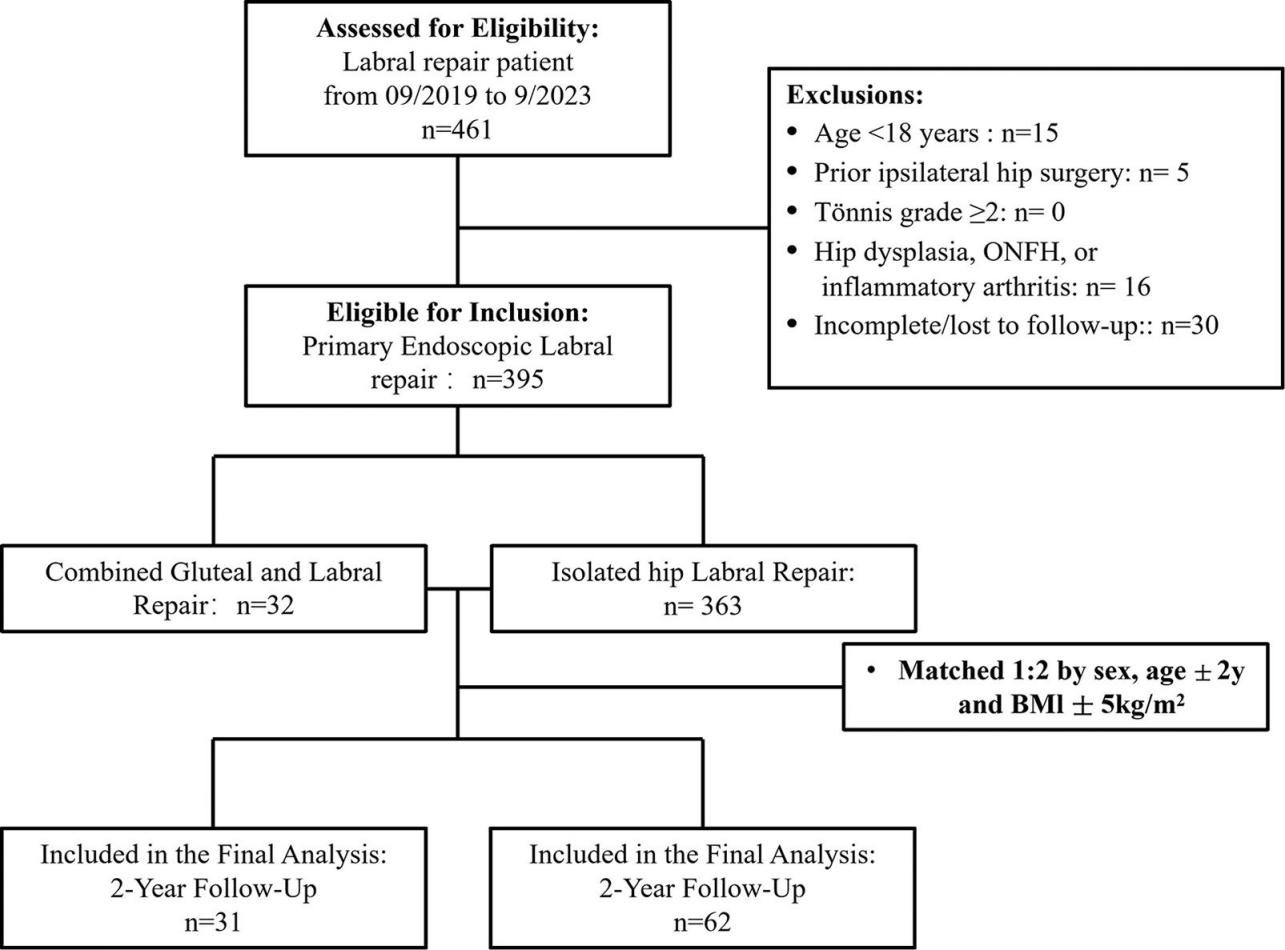
CONSORT flowchart for patient selection. CONSORT, Consolidated Standards of Reporting Trials; ONFH, osteonecrosis of the femoral head.

The study protocol was approved by the Institutional Review Board of Second Qilu Hospital of Shandong University (approval No. LCLL-2022–003). This retrospective study consecutively reviewed patients who underwent primary hip arthroscopy between September 2019 and September 2023, all of whom were evaluated and surgically treated by a single senior arthroscopic surgeon (Q.F.Y.). Patients were categorized into two cohorts: a combined repair group, comprising patients with acetabular labral tears and concomitant hip rotator cuff injuries, and an isolated labral repair group, comprising patients with labral tears only. All patients underwent standardized preoperative evaluations in the outpatient clinic, including detailed medical history acquisition, focused physical examination, and comprehensive imaging assessment. The physical examination documented lateral hip pain, nocturnal pain, tenderness over the greater trochanter, and the presence of a positive Trendelenburg sign. In addition, resisted hip flexion and internal rotation testing, lateral stair climbing test, and flexion–adduction–internal rotation test were performed. Imaging assessment consisted of standing anteroposterior pelvic radiographs, Dunn view radiographs, false-profile radiographs, and unilateral hip magnetic resonance imaging (MRI). All clinical diagnoses were independently confirmed by the operating surgeon. The inclusion criteria were as follows: (1) acetabular labral tears associated with femoroacetabular impingement syndrome (FAIS) confirmed by MRI and physical examination, with concomitant hip rotator cuff injuries required for inclusion in the combined repair group (Figure 1); (2) failure of structured nonoperative management; (3) availability of complete operative records; and (4) a minimum postoperative follow-up duration of 2 years. Exclusion criteria included: (1) age younger than 18 years; (2) a history of prior surgery on the ipsilateral hip; (3) radiographic evidence of hip osteoarthritis with a Tönnis grade ≥2; (4) coexisting hip dysplasia, osteonecrosis of the femoral head, or other inflammatory joint diseases; and (5) incomplete follow-up data or loss to follow-up. To minimize potential confounding bias, propensity scores were estimated using logistic regression, and nearest-neighbor matching was performed. Patients in the combined repair group were matched to those in the isolated labral repair group at a 1:2 ratio. Patients in the isolated labral repair group were confirmed by the same surgeon (Q.F.Y.) to have acetabular labral lesions based on MRI and physical examination, with no evidence of gluteal tendon pathology. Matching variables included sex, age (±2 years), and body mass index (BMI; ±5 kg/m^2^). All matched patients met the same inclusion and exclusion criteria described above. A detailed flowchart of patient selection is presented in Figure 2.

### 2.2 Preoperative and intraoperative data collection

Baseline demographic characteristics, including sex, age, body mass index (BMI), operative side, and follow-up duration, were obtained from the institutional electronic medical records. Intraoperative variables included the extent and pattern of gluteal tendon tears (categorized as partial- or full-thickness tears involving the gluteus medius, gluteus minimus, or both), the number of suture anchors utilized, and the performance of femoroplasty or acetabuloplasty. Patient-reported outcomes (PROs) were evaluated using standardized, validated instruments. All PRO measures were collected preoperatively and at a minimum of 2 years postoperatively. Outcome measures included the modified Harris Hip Score (mHHS), the 12-item International Hip Outcome Tool (iHOT-12), the Western Ontario and McMaster Universities Osteoarthritis Index (WOMAC), and the visual analog scale (VAS) for pain and patient satisfaction.

### 2.3 Surgical technique

All procedures were performed under total intravenous anesthesia (TIVA). Patients were positioned supine on a traction table without use of a perineal post. The operative limb was placed in approximately 5°–10° of hip flexion, 15° of abduction, and 45° of internal rotation, a position that facilitates relaxation of the anterior capsule and improves access to the peritrochanteric space. The contralateral limb was secured in 45° of abduction.

Preoperatively, bony landmarks were marked on the skin, including the anterior superior iliac spine and the apex and contours of the greater trochanter. Standard arthroscopic portals for both intra-articular and peritrochanteric procedures of the hip were utilized. The anterolateral (AL) portal, located approximately 1 cm anterior and superior to the tip of the greater trochanter, was established as the initial viewing portal. The mid-anterior (MA) portal was created under direct arthroscopic visualization approximately 5 cm distal and medial to the AL portal to ensure safe access to the joint. The distal anterolateral accessory (DALA) portal was established approximately 10 cm distal to the AL portal along the longitudinal axis of the femur and served as the primary working portal for peritrochanteric evaluation and intervention.

A sequential three-step arthroscopic strategy was employed, consisting of exploration and assessment of the peritrochanteric space, management of intra-articular labral pathology, and repair of hip rotator cuff injuries involving the gluteus medius and/or gluteus minimus tendons. The procedure began with inspection of the peritrochanteric space. Trochanteric bursectomy was performed, followed by systematic identification of relevant anatomic landmarks. The vastus lateralis tendon, which appeared as a bright and well-defined tendinous structure at its insertion on the greater trochanter, was readily identifiable arthroscopically and served as a reliable reference for localization of the gluteus medius and gluteus minimus tendons. The gluteus medius insertion was located proximal and posterior to the vastus lateralis, whereas the gluteus minimus insertion was located proximal and anterior. The lateral and anterior facets of the greater trochanter were thoroughly inspected to assess tendon integrity.

**Figure 3:**
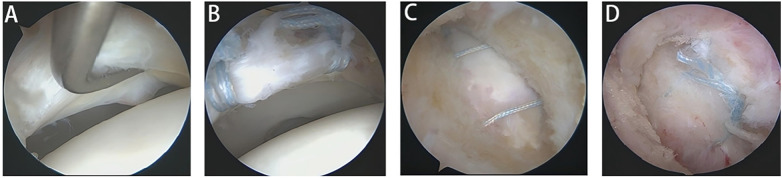
Exploration and repair of acetabular labral tears. **(A)** Exploration reveals labral tear. **(B)** Labral suture. **(C,D)** Side-to-side suture closure of joint capsule.

Following evaluation of the peritrochanteric space, attention was directed to the intra-articular compartment for labral management. A longitudinal outside-in capsulotomy was performed to expose the peripheral compartment and acetabular labrum. Under direct arthroscopic visualization, longitudinal traction was applied to distract the joint and allow inspection of the central compartment. Labral tears associated with instability were treated with suture anchor–based repair, whereas labral lesions with acceptable stability were managed with selective debridement and radiofrequency-assisted stabilization. Concomitant chondral lesions, ligamentum teres pathology, or loose bodies were addressed as indicated. After completion of peripheral compartment procedures with the hip in flexion, the capsule was routinely closed in a side-to-side fashion using interrupted nonabsorbable sutures (Figure 3).

**Figure 4:**
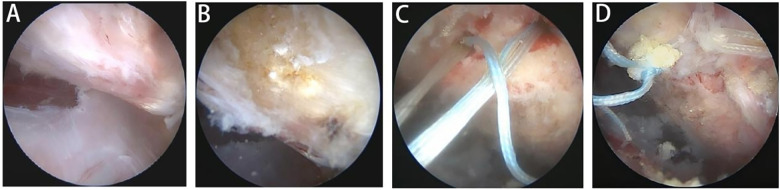
Arthroscopic exploration and repair of rotator cuff tears in the Hip joint. **(A)** Arthroscopy reveals a full-thickness tear of the gluteus medius muscle with a distinct tendon rupture. **(B)** Arthroscopy shows hypercalcified lesions in the greater trochanter region. **(C)** Prepare the bone bed by debriding and implanting anchor screws. **(D)** Perform single-row screw fixation.

After completion of labral management, the procedure returned to the peritrochanteric space for definitive treatment of hip rotator cuff injuries. The repair strategy was determined based on the severity of tendon injury. Tendinopathic changes were treated with radiofrequency ablation, whereas partial- or full-thickness tendon tears were repaired using suture anchors. Prior to tendon fixation, the greater trochanteric footprint was prepared by debriding calcified tissue while preserving viable tendon substance, followed by decortication of the bony surface using a shaver or burr. After anchor placement, sutures were passed using a suture hook. Depending on tear configuration and footprint coverage, either single-row fixation or double-row suture-bridge fixation was performed (Figure 4).

### 2.4 Postoperative outcomes

Patient-reported outcomes were prospectively collected and retrospectively analyzed at the 2-year postoperative follow-up. The assessment tools utilized included the mHHS, iHOT-12, WOMAC, as well as VAS for pain and the VAS for patient satisfaction. To evaluate the clinical significance of postoperative improvements, the minimal clinically important difference (MCID) and patient acceptable symptom state (PASS) were employed as threshold-based outcome measures. The cutoff values for MCID and PASS were derived from previously published studies examining two-year outcomes following arthroscopic labral repair and were defined as follows: mHHS (MCID: 9.2; PASS: 83.25), iHOT-12 (MCID: 13.9; PASS: 72.2), VAS for pain (MCID: 14.8; PASS: 21.6), and VAS for satisfaction (PASS: 80.9) (9).

### 2.5 Postoperative rehabilitation

All patients in both groups followed a standardized three-phase postoperative rehabilitation protocol. During the early postoperative phase (weeks 1–6), patients wore a hip brace to restrict active abduction. Partial weight-bearing was permitted with assistive devices, along with limited passive range-of-motion exercises. During the intermediate phase (weeks 6–12), patients progressed gradually to full weight-bearing; progressive hip muscle strengthening exercises were introduced, and the hip brace was discontinued. During the late phase (week 12 onward), the rehabilitation program focused on restoring independent ambulation and facilitating a gradual return to activities of daily living. Completion of the standardized rehabilitation protocol was anticipated by approximately 24 weeks postoperatively.

### 2.6 Statistical analysis

Continuous variables were reported as mean ± standard deviation and were compared between groups using two-tailed independent-samples t-tests. Preoperative and 2-year postoperative patient-reported outcomes were compared using paired-samples t-tests. Categorical variables were reported as frequency (percentage) and were compared between groups using Pearson's chi-square or Fisher's exact test. An *a priori* power analysis was performed with an alpha level of 0.05 and a power of 80%. Assuming a 1:2 matching ratio, a sample size of 28 cases was determined to be necessary for detecting differences in mHHS. An alpha level of 0.05 was used to determine statistical significance for all tests. All statistical analyses were performed using SPSS (Version 28.0; IBM Corp., Armonk, NY, USA).

## 3 Results

### 3.1 Patient demographics

A total of 31 patients who underwent simultaneous arthroscopic repair of hip rotator cuff injuries and acetabular labral tears were included in the study (Figure 2). These 31 patients (26 women and 5 men; mean age, 52.13 ± 7.47 years; BMI, 28.33 ± 5.42) were matched at a 1:2 ratio based on sex, age, and BMI with 62 patients who underwent isolated arthroscopic labral repair (52 women and 10 men; mean age, 51.97 ± 7.21 years; mean BMI, 28.50 ± 5.35). No significant differences were observed between the two groups with respect to sex (*P* > 0.999), age (*P* = 0.920), or BMI (*P* = 0.886), indicating that the groups were well matched and comparable (Table 1).

**Table 1:** Patient demographic data.

Characteristic	Gluteal and labral repair	Labral repair only	*P*
No. of patients	31	62	
Age, mean ± SD	52.13 ± 7.47	51.97 ± 7.21	0.920
Sex, n			>0.999
Male	5 (16.13)	10 (16.13)	
Female	26 (83.87)	52 (83.87)	
BMI, mean ± SD	28.33 ± 5.42	28.50 ± 5.35	0.886
Laterality			0.769
Left	16 (51.61)	34 (54.84)	
Right	15 (48.39)	28 (45.16)	
Follow-up time, mo	32.96 ± 4.97	33.92 ± 4.66	0.363

### 3.2 Preoperative clinical and imaging characteristics

Regarding clinical symptoms, all patients in the combined hip rotator cuff and labral repair group reported lateral hip pain, compared with 8.1% of patients in the isolated labral repair group. Night pain was reported in 45.2% of patients in the combined repair group and in 16.1% of patients in the isolated labral repair group, with a statistically significant between-group difference (*P* < 0.001). Tenderness over the greater trochanter was present in all patients in the combined repair group, compared with 9.7% of patients in the labral repair group. A positive Trendelenburg sign was observed in 64.5% of patients in the combined repair group and in 1.6% of patients in the labral repair group, with a statistically significant difference between groups (*P* < 0.001). Detailed physical examination findings are summarized in Table 2. In the combined repair group, the rates of positive findings on the resisted internal rotation in hip flexion, lateral stair climbing test, and flexion-adduction-internal rotation were 77.4%, 90.3%, and 83.9%, respectively. Compared with the isolated labral repair group, significantly higher positive rates were observed for the resisted internal rotation test and the lateral stair climbing test (both *P* < 0.001), whereas no significant difference was found in the rate of a positive flexion-adduction-internal rotation (*P* = 0.570). All patients underwent preoperative hip magnetic resonance imaging. In the combined repair group, MRI demonstrated partial-thickness hip rotator cuff tears in 87.1% of patients and full-thickness tears in 12.9% (Table 4). No significant differences were identified between groups with respect to radiographic parameters, including the LCEA (*P* = 0.258), ACEA (*P* = 0.199), Tönnis angle (*P* > 0.999), neck-shaft angle (*P* = 0.712) and alpha angle (*P* = 0.338) (Table 3).

**Table 2:** Patient clinical characteristics data.

Characteristic	Gluteal and labral repair	Labral repair only	*P*
Lateral hip pain	100	8.1	<0.001
Nighttime pain	45.2	16.1	<0.001
Greater trochanter tenderness	100	9.7	<0.001
Limping	25.8	8.1	0.045
Trendelenburg sign	64.5	1.6	<0.001
Resisted Internal Rotation (Flexion)	77.4	0	<0.001
Lateral stair climbing test	90.3	0	<0.001
Flexion Adduction Internal Rotation test	83.9	90.3	0.570

**Table 3:** Preoperative radiographic characteristics data.

Variable	Gluteal and labral repair	Labral repair only	*P*	95%CI	d
Dunn alpha angle, deg	58.36 ± 5.37	59.50 ± 5.32	0.338	−3.46 to 1.20	0.212
LCEA, deg	31.46 ± 5.17	30.15 ± 5.22	0.258	−0.97 to 3.58	0.250
ACEA, deg	32.41 ± 4.40	30.90 ± 5.69	0.199	−0.81 to 3.83	0.280
Tönnis grade			>0.999		
0	26 (83.87)	52 (83.87)			
1	5 (16.13)	10 (16.13)			
Neck-shaft angle	131.53 ± 5.06	131.15 ± 4.37	0.712	−1.64 to 2.39	0.082

**Table 4:** Intraoperative findings and procedures.

Variable	Gluteal and labral repair	Labral repair only	*P*
Extent of gluteus tearing, %			
Full thickness	12.9		
Partial thickness	87.1		
Gluteus tendons involved, %			
Gluteus minimus	9.7		
Gluteus medius	38.4		
Both	51.9		
No. of anchors			
Labrum	1.9 ± 0.5	1.9 ± 0.5	0.941
Gluteus	1.8 ± 0.4		
Femoroplasty, %	93.5	91.9	0.781
Acetabuloplasty, %	87.1	88.7	0.820

### 3.3 Intraoperative findings and procedures

**Figure 5:**
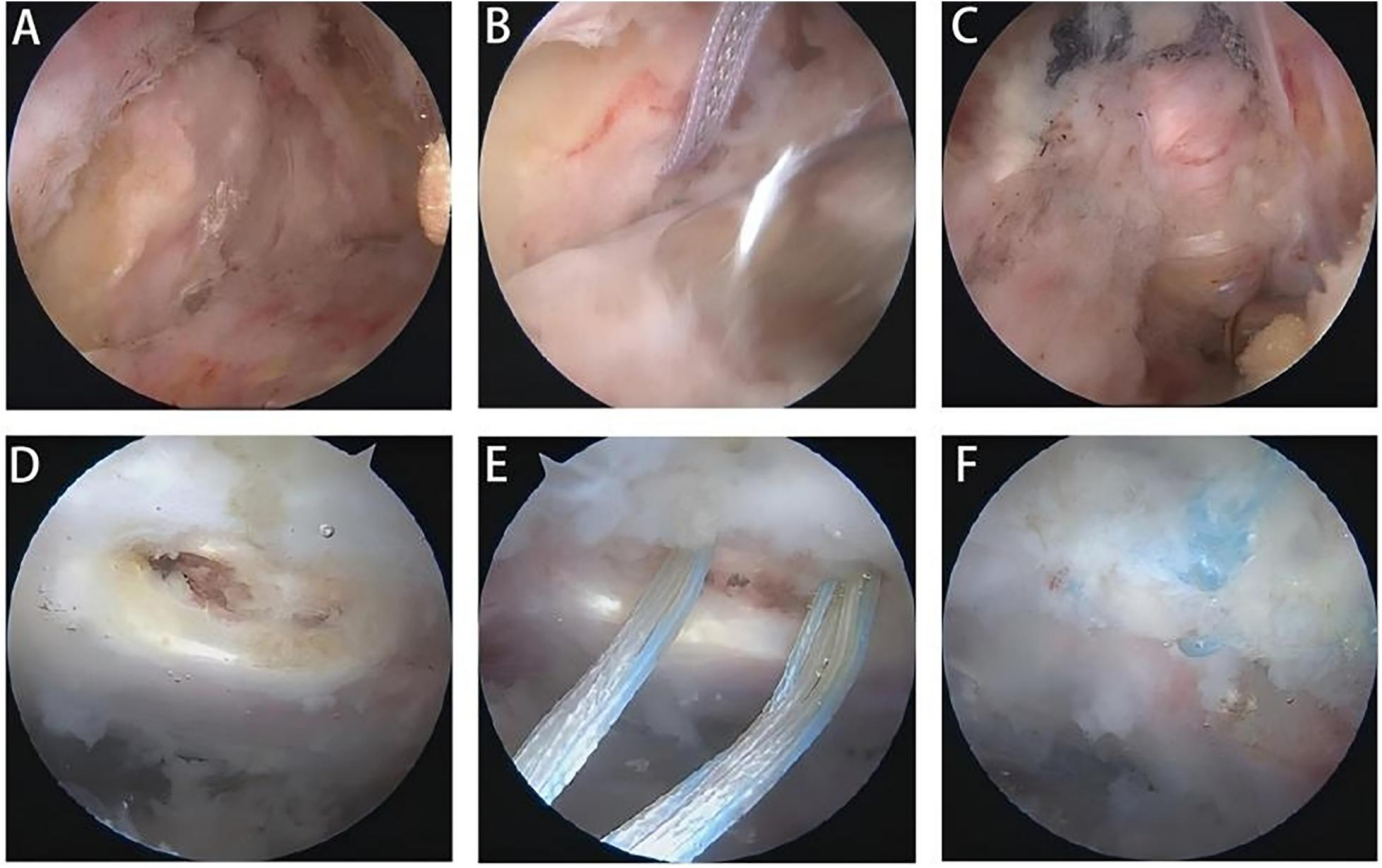
Typical images of full-thickness and partial repair of gluteus medius tears. **(A)** Exploration reveals a full-thickness tear of the gluteus medius muscle. **(B)** Debridement of the bone bed and anchor implantation. **(C)** Suture repair of the gluteus medius muscle. **(D)** Exploration reveals a partial tear of the gluteus medius muscle. **(E)** Debridement of the bone bed and anchor implantation. **(F)** Suture repair of the gluteus medius muscle.

No significant differences were observed between groups regarding the presence or severity of gluteus medius and/or gluteus minimus tendon pathology, including the proportion of partial- vs. full-thickness tears. Among patients undergoing combined hip rotator cuff and labral repair, concomitant involvement of both the gluteus medius and gluteus minimus tendons was most common (16 patients, 51.9%), followed by isolated gluteus medius involvement (12 patients, 38.4%). Isolated gluteus minimus involvement was relatively uncommon (3 patients, 9.7%). Partial-thickness tears were significantly more prevalent than full-thickness tears (87.1% vs. 12.9%) (Figure 4). The number of suture anchors used for labral repair did not differ significantly between groups (*P* = 0.941) (Figure 5). In addition, the majority of patients in the combined repair group underwent concomitant correction of femoroacetabular impingement morphology, with femoroplasty and acetabuloplasty performed in 93.5% and 87.1% of patients, respectively. These rates were comparable to those observed in the isolated labral repair group, with no significant between-group differences (femoroplasty rate: *P* = 0.781; acetabuloplasty rate: *P* = 0.820) (Table 4).

### 3.4 Postoperative outcomes

Both groups demonstrated significant improvements from preoperatively to 2-year follow-up in all patient-reported outcome measures, including the mHHS, iHOT-12, WOMAC, and VAS for pain (all *P* < 0.001). At both the preoperative assessment and the 2-year follow-up, no significant differences were observed between the combined repair group and the isolated labral repair group with respect to mHHS, iHOT-12, WOMAC, VAS pain, or VAS satisfaction scores (all *P* > 0.05) (Table 5). Regarding clinically meaningful improvement, the proportions of patients in the combined repair group achieving the MCID and/or PASS were as follows: mHHS (83.9%/38.7%), iHOT-12 (77.4%/45.2%), VAS pain (87.1%/61.3%), and VAS satisfaction (64.5%). These rates did not differ significantly from those observed in the isolated labral repair group (all *P* > 0.05) (Table 6).

**Table 5:** Preoperative and 2-year postoperative in PRO scores.

PRO	Gluteal and Labral Repair	Labral Repair only	*P*	95%CI	d
Preoperative					
No. of patients	31	62			
mHHS	50.48 ± 10.04	53.76 ± 8.66	0.107	−7.26 to 0.72	0.358
iHOT-12	37.70 ± 10.28	40.97 ± 9.50	0.131	−7.53 to 1.00	0.335
WOMAC	17.59 ± 10.39	18.01 ± 10.03	0.851	−4.85 to 4.01	0.041
VAS pain	61.38 ± 10.47	64.40 ± 10.65	0.198	−7.55 to 1.74	0.273
2-y postoperative					
No. of patients	31	62			
mHHS	79.09 ± 8.09	79.70 ± 7.26	0.714	−3.91 to 2.69	0.081
iHOT-12	69.43 ± 9.11	72.70 ± 8.58	0.092	−7.10 to 0.55	0.374
WOMAC	10.76 ± 4.55	11.28 ± 5.91	0.672	−2.91 to 1.89	0.093
VAS pain	23.13 ± 9.45	23.71 ± 7.58	0.750	−4.18 to 3.02	0.070
VAS satisfaction	82.84 ± 7.66	84.47 ± 6.58	0.289	−4.67 to 1.41	0.235

PRO, patient-reported outcome; mHHS, modified Harris Hip Score; iHOT-12,12-tem International Hip Outcome Tool; WOMAC, Western Ontario and McMaster Universities Osteoarthritis Index; VAS, visual analog scale.

**Table 6:** Achievement rates of MCID and PASS.

Variable	Gluteal and labral repair	Labral repair only	*P*
MCID			
No. of patients	31	62	
mHHS	83.9	82.3	0.846
iHOT-12	77.4	83.9	0.448
VAS pain	87.1	85.5	0.833
PASS			
No. of patients	31	62	
mHHS	38.7	37.1	0.880
iHOT-12	45.2	48.4	0.769
VAS pain	61.3	59.7	0.881
VAS satisfaction	64.5	69.4	0.638

mHHS, modified Harris Hip Score; iHOT-12,12-tem International Hip Outcome Tool; VAS, visual analog scale.

### 3.5 Secondary surgeries and complications

No significant differences were observed between groups in the rates of postoperative complications, revision hip arthroscopy, and conversion to total hip arthroplasty (all *P* > 0.05). In the combined repair group, 1 patient (3.2%) underwent revision surgery at 24 months postoperatively because of a retear of the gluteus medius tendon. In the labral repair group, 1 patient (1.6%) underwent conversion to total hip arthroplasty at 28 months postoperatively. A total of 8 patients experienced postoperative anterolateral thigh hypoesthesia consistent with lateral femoral cutaneous nerve irritation, including 3 patients in the combined repair group and 5 patients in the labral repair group. All affected patients were treated with mecobalamin, and symptoms resolved by the 6-month follow-up. No cases of postoperative infection, trochanteric bursitis, or deep vein thrombosis were observed in either group (Table 7).

**Table 7:** The rate of revision, and conversion to THA and postoperative complications.

Variable	Gluteal and labral repair	Labral repair only	*P*
No. of patients	31	62	
Future surgery			
Revision	3.2	0	0.333
THA	0	1.6	>0.999
Complications			
Postoperative infection	0	0	
Trochanteric bursitis	0	0	
Deep vein thrombosis	0	0	
Lateral femoral cutaneous nerve injury	9.7	8.1	0.794

THA, total hip arthroplasty.

## 4 Discussion

The principal finding of this 2-year propensity score–matched cohort study was that patients with concomitant acetabular labral tears and hip rotator cuff injuries (gluteus medius and/or gluteus minimus tendon pathology) who underwent simultaneous arthroscopic repair showed no statistically significant difference in short-term clinical outcomes compared to those treated with isolated arthroscopic labral repair. At the 2-year follow-up, all patients demonstrated significant improvements from baseline across multiple patient-reported outcome measures, with no significant differences between groups in final PRO scores or in the proportions of patients achieving the MCID and PASS. This indicates that the concurrent gluteal tendon repair did not adversely affect the early prognosis of the labral repair.

From a demographic perspective, patients undergoing combined labral and hip rotator cuff repair in the present study had a mean age of 52.1 years, which was notably higher than the mean age of patients in our institutional database undergoing isolated labral repair (41.5 years). These findings indicate that concomitant hip rotator cuff injuries are more frequently encountered in middle-aged and older patients undergoing labral repair. This observation is consistent with prior epidemiologic studies demonstrating a marked age-related increase in the prevalence of hip rotator cuff injuries, particularly among individuals aged 40 to 60 years (10). In addition, female patients accounted for 83.9% of the combined repair cohort, suggesting a substantially higher risk in women (11, 12). This sex-based disparity may be multifactorial. A wider pelvic morphology in women may increase the abductor lever arm and mechanical stress on the gluteus medius tendon, while increased pelvic tilt may alter hip biomechanics and exacerbate abductor tendon loading. Furthermore, postmenopausal declines in estrogen levels may adversely affect tendon collagen metabolism and tissue quality, predisposing tendons to degeneration and injury (13–16).

Symptoms and physical examination findings also differed distinctly between groups. All patients with combined hip rotator cuff and labral pathology reported lateral hip pain, whereas this symptom was present in only 8.1% of patients with isolated labral tears. Night pain was also significantly more common in the combined repair group. On physical examination, tenderness over the greater trochanter was present in all patients with concomitant rotator cuff pathology but was uncommon in patients with isolated labral tears. Collectively, these findings suggest that lateral hip pain, nocturnal pain, and greater trochanteric tenderness represent important clinical indicators of underlying hip rotator cuff injuries.

However, the overlapping symptomatology poses diagnostic challenges. Anterior groin pain, typically associated with labral pathology or femoroacetabular impingement syndrome (FAIS), was common in both groups, and the rate of a positive flexion-adduction-internal rotation did not differ between groups. This finding underscores the high sensitivity but limited specificity of flexion-adduction-internal rotation. Diagnostic complexity is further compounded in older patients by the potential coexistence of age-related degenerative labral changes and primary or secondary hip rotator cuff injuries. In contrast, the Trendelenburg sign demonstrated a significant between-group difference, consistent with classic biomechanical theory that a positive Trendelenburg sign reflects substantial abductor weakness (17). Notably, the resisted internal rotation test in hip flexion and the lateral stair climbing test showed particularly high discriminatory value, with significantly higher positive rates in the combined repair group. These findings support the utility of these maneuvers as targeted clinical tools for identifying hip rotator cuff injuries in patients presenting with labral tears (18, 19).

Beyond clinical assessment, imaging is critical in establishing the diagnosis. Radiographic findings such as greater trochanteric enthesophytes may serve as indirect indicators of abductor tendinopathy (11). Magnetic resonance imaging, particularly with dedicated hip protocols or unilateral hip MRI, remains the gold standard for evaluating soft tissue pathology (20–23). In the present study, all patients undergoing combined repair demonstrated MRI evidence of hip rotator cuff injuries preoperatively, which was subsequently confirmed intraoperatively. Importantly, no differences were observed between groups in osseous morphologic parameters associated with FAIS, including the lateral center-edge angle, Tönnis angle, and alpha angle. This finding suggests that while FAIS-related bony morphology may represent a shared biomechanical background, it does not distinguish between isolated labral pathology and combined labral–rotator cuff injury. Whether hip rotator cuff injuries represent a compensatory response to altered hip biomechanics following labral injury or constitutes a primary coexisting disorder remains unclear and warrants further investigation. Nevertheless, the present study confirms that these pathologies frequently coexist in clinical practice.

Optimal surgical management of patients with combined labral and hip rotator cuff injuries remains controversial. While staged procedures may increase patient burden and healthcare costs, simultaneous repair presents its own technical challenges related to operative time, tissue edema, and visualization. Surgical sequencing strategies vary. Horner et al. reported performing intra-articular labral repair and osseous correction before addressing hip rotator cuff injuries with the hip in abduction (18). In contrast, the present study employed and validated a sequential three-step approach consisting of initial peritrochanteric space exploration, followed by intra-articular labral management, and concluding with definitive hip rotator cuff repair. This sequential strategy offers several advantages. First, initiating the procedure in the peritrochanteric space allows assessment and preparation of the abductor footprint before capsulotomy, thereby minimizing fluid extravasation and soft tissue edema and providing optimal visualization for tendon evaluation and anchor placement. Second, performing rotator cuff repair after completion of intra-articular procedures minimizes traction-related stress on the repaired abductor tendons. Third, the supine position facilitates seamless transition between intra-articular and extra-articular procedures without the need for specialized traction setups, thereby streamlining the surgical workflow.

Regarding repair technique, secure tendon-to-bone fixation is the cornerstone of successful hip rotator cuff repair. Drawing from principles established in shoulder rotator cuff surgery, both single-row and double-row suture-bridge constructs were used based on tear size, configuration, tendon quality, and degree of retraction. Larger or retracted tears with compromised tissue quality may benefit from double-row fixation, which provides greater footprint coverage and improved biomechanical strength and may reduce retear risk (24). However, important anatomic and biomechanical differences exist between the hip and shoulder, and the optimal fixation strategy for hip rotator cuff repair remains to be definitively established (25, 26). In the present study, patients undergoing combined repair achieved pain relief, functional improvement, and satisfaction scores comparable to those of patients undergoing isolated labral repair, without an increased rate of complications, demonstrating the safety and feasibility of this combined approach.

An additional unresolved question is whether routine intra-articular inspection is necessary in patients undergoing hip rotator cuff repair. In the present cohort, all patients undergoing combined repair had concomitant labral pathology that was addressed arthroscopically, consistent with prior reports of a high prevalence of labral lesions in patients with hip rotator cuff injuries (27, 28). Failure to address symptomatic intra-articular pathology at the time of abductor repair has been associated with suboptimal postoperative outcomes (29). Therefore, we recommend routine intra-articular evaluation during arthroscopic treatment of hip rotator cuff injuries, with simultaneous repair of clinically relevant labral lesions when identified.

Overall, the most salient finding of this study is that simultaneous arthroscopic repair of concomitant hip rotator cuff and labral pathology does not compromise clinical outcomes. Both groups demonstrated significant improvements in mHHS, iHOT-12, WOMAC, and VAS pain scores, with no between-group differences in postoperative outcomes or in rates of achieving MCID and PASS. Complication rates were low, further supporting the short-term safety of this strategy.

## 5 Limitations

Several limitations should be acknowledged. First, the retrospective design inherently carried a risk of selection bias, although propensity score matching was applied to mitigate this concern. Second, the sample size was limited, which may have reduced our ability to perform adequately powered subgroup analyses and to detect true between-group differences in rare adverse events, such as deep vein thrombosis or surgical site infection. Third, the relatively short follow-up precluded evaluation of long-term outcomes, progression of osteoarthritis, and retear rates. Future multicenter, prospective studies with larger cohorts and longer follow-up are needed to further substantiate the safety of concurrent repair and to refine surgical strategies.

## 6 Conclusion

This propensity score–matched study demonstrates that a sequential arthroscopic strategy involving simultaneous labral repair and hip rotator cuff repair is safe, feasible, and effective for patients with concomitant acetabular labral tears and hip rotator cuff injuries. At 2-year follow-up, patients undergoing combined repair achieved short-term clinical outcomes comparable to those undergoing isolated labral repair, indicating that the addition of gluteal tendon repair did not impair the early outcomes of labral repair. These findings provide important clinical evidence supporting simultaneous arthroscopic repair as a viable treatment option for patients with combined labral and hip rotator cuff injuries.

## Data Availability

The dataset contains sensitive patient-level information and is not publicly available due to institutional ethics approval requirements and privacy regulations. De-identified data may be made available from the corresponding author upon reasonable request and with approval from the Institutional Ethics Review Committee of the Second Hospital of Shandong University. Requests to access the datasets should be directed to 2993356033@qq.com.

## References

[1] Groh MM Herrera J. A comprehensive review of hip labral tears. Curr Rev Musculoskelet Med. (2009) 2(2):105–17. 10.1007/s12178-009-9052-9 19468871 PMC2697339

[2] Chi AS Long SS Zoga AC Read PJ Deely DM Parker L Prevalence and pattern of gluteus medius and minimus tendon pathology and muscle atrophy in older individuals using mri. Skeletal Radiol. (2015) 44(12):1727–33. 10.1007/s00256-015-2220-7 26260535

[3] Lee JW Hwang DS Kang C Hwang JM Chung HJ. Arthroscopic repair of acetabular labral tears associated with femoroacetabular impingement: 7–10 years of long-term follow-up results. Clin Orthop Surg. (2019) 11(1):28–35. 10.4055/cios.2019.11.1.28 30838105 PMC6389536

[4] Vassalo CC Barros AAG Costa LP Guedes EC de Andrade MAP. Clinical outcomes of arthroscopic repair of acetabular labral tears. BMJ Open Sport Exerc Med. (2018) 4(1):e000328. 10.1136/bmjsem-2017-000328 29862041 PMC5976113

[5] Byrd JW Jones KS. Primary repair of the acetabular labrum: outcomes with 2 Years’ follow-up. Arthroscopy. (2014) 30(5):588–92. 10.1016/j.arthro.2014.02.007 24725313

[6] Griffin DR Parsons N Mohtadi NG Safran MR. A short version of the international hip outcome tool (Ihot-12) for use in routine clinical practice. Arthroscopy. (2012) 28(5):611–6; quiz 6–8. 10.1016/j.arthro.2012.02.027 22542434

[7] Hartigan DE Perets I Ho SW Walsh JP Yuen LC Domb BG. Endoscopic repair of partial-thickness undersurface tears of the abductor tendon: clinical outcomes with minimum 2-year follow-up. Arthroscopy. (2018) 34(4):1193–9. 10.1016/j.arthro.2017.10.022 29305287

[8] Thaunat M Chatellard R Noël E Sonnery-Cottet B Nové-Josserand L. Endoscopic repair of partial-thickness undersurface tears of the gluteus medius tendon. Orthop Traumatol Surg Res. (2013) 99(7):853–7. 10.1016/j.otsr.2013.06.005 24075011

[9] Horner NS Chapman RS Larson JH Nho SJ. Results of endoscopic labral repair with concomitant gluteus medius and/or minimus repair compared with outcomes of labral repair alone: a matched comparative cohort analysis at Minimum 2-year follow-up. Am J Sports Med. (2023) 51(7):1818–25. 10.1177/03635465231166708 37103484

[10] Howell GE Biggs RE Bourne RB. Prevalence of abductor mechanism tears of the hips in patients with osteoarthritis. J Arthroplasty. (2001) 16(1):121–3. 10.1054/arth.2001.19158 11172282

[11] Walsh MJ Walton JR Walsh NA. Surgical repair of the gluteal tendons: a report of 72 cases. J Arthroplasty. (2011) 26(8):1514–9. 10.1016/j.arth.2011.03.004 21798694

[12] LaPorte C Vasaris M Gossett L Boykin R Menge T. Gluteus medius tears of the hip: a comprehensive approach. Phys Sportsmed. (2019) 47(1):15–20. 10.1080/00913847.2018.1527172 30244629

[13] Connell DA Bass C Sykes CA Young D Edwards E. Sonographic evaluation of gluteus medius and minimus tendinopathy. Eur Radiol. (2003) 13(6):1339–47. 10.1007/s00330-002-1740-4 12764651

[14] SL B. Systematic Anatomy[M]. 7th edn. Beijing: People's Medical Publishing House (2008). p. 52–4.

[15] Liang T Wu YK Yin QF. Research progress in the mechanism and clinical diagnosis and treatment of rotator cuff injury of hip joints. Chin J New Clin Med. (2020) 13(6):555–9. 10.3969/j.issn.1674-3806.2020.06.04

[16] Ganderton C Semciw A Cook J Pizzari T. Does menopausal hormone therapy (Mht), exercise or a combination of both, improve pain and function in post-menopausal women with greater trochanteric pain syndrome (Gtps)? A randomised controlled trial. BMC Womens Health. (2016) 16:32. 10.1186/s12905-016-0311-9 27312538 PMC4910216

[17] Bird PA Oakley SP Shnier R Kirkham BW. Prospective evaluation of magnetic resonance imaging and physical examination findings in patients with greater trochanteric pain syndrome. Arthritis Rheum. (2001) 44(9):2138–45. 10.1002/1529-0131(200109)44:9<2138::Aid-art367>3.0.Co;2-m 11592379

[18] Walker-Santiago R Ortiz-Declet V Maldonado DR Wojnowski NM Domb BG. The modified resisted internal rotation test for detection of gluteal tendon tears. Arthrosc Tech. (2019) 8(3):e331–e4. 10.1016/j.eats.2018.11.006 31019887 PMC6471346

[19] Ross M. Test-Retest reliability of the lateral step-up test in young adult healthy subjects. J Orthop Sports Phys Ther. (1997) 25(2):128–32. 10.2519/jospt.1997.25.2.128 9007771

[20] Yang S Xie Q Su F Wu YT Yang YT. Progress in mri evaluation of hip development after closed reduction for developmental dysplasia of the hip. Chin J Orthop. (2022) 42(8):538–44. 10.3760/cma.j.cn121113-20210718-00460

[21] Kingzett-Taylor A Tirman PF Feller J McGann W Prieto V Wischer T Tendinosis and tears of gluteus medius and minimus muscles as a cause of hip pain: mr imaging findings. AJR Am J Roentgenol. (1999) 173(4):1123–6. 10.2214/ajr.173.4.10511191 10511191

[22] Westacott DJ Minns JI Foguet P. The diagnostic accuracy of magnetic resonance imaging and ultrasonography in gluteal tendon tears–a systematic review. Hip Int. (2011) 21(6):637–45. 10.5301/hip.2011.8759 22038311

[23] Tian CY Y H Wang JQ. 3.0 T high-resolution Mri of ace- tabular labrum tear. Diagn Imaging Interv Radiol. (2016) 2016(2):138–41. 10.3969/j.issn.1005-8001.2016.02.010

[24] Kahlenberg CA Nwachukwu BU Jahandar H Meyers KN Ranawat AS Ranawat AS. Single- vs. double-row repair of hip abductor tears: a biomechanical matched cadaver study. Arthroscopy. (2019) 35(3):818–23. 10.1016/j.arthro.2018.10.146 30733037

[25] Yuan B Tian M Zhang SL Ma D Li YM Zeng JJ. Predictive value of shoulder joint anatomical features to the small and Medium rotator cuff retear rate after rehabilitation. Chin J Orthop. (2023) 43(18):1193–200. 10.3760/cma.j.cn121113-20230519-00292

[26] Jiang JS Yu XY Dong YS Wang SF. Research methods and progress of gradient multiphase scaffold materials promoting rotator cuff tenon-bone interface repair and regeneration. Chin J Orthop. (2023) 43(14):991–8. 10.3760/cma.j.cn121113-20221206-00692

[27] Byrd JWT Jones KS. Endoscopic repair of hip abductor tears: outcomes with two-year follow-up. J Hip Preserv Surg. (2017) 4(1):80–4. 10.1093/jhps/hnw047 28630725 PMC5467421

[28] Perets I Mansor Y Yuen LC Chen AW Chaharbakhshi EO Domb BG. Endoscopic gluteus medius repair with concomitant arthroscopy for labral tears: a case series with Minimum 5-year outcomes. Arthroscopy. (2017) 33(12):2159–67. 10.1016/j.arthro.2017.06.032 28969951

[29] Meghpara MB Yelton MJ Glein RM Malik MS Rosinsky PJ Shapira J Isolated endoscopic gluteus medius repair can achieve successful clinical outcomes at Minimum 2-year follow-up. Arthrosc Sports Med Rehabil. (2021) 3(6):e1697–e704. 10.1016/j.asmr.2021.07.026 34977622 PMC8689210

